# Full length talin stimulates integrin activation and axon regeneration

**DOI:** 10.1016/j.mcn.2015.03.011

**Published:** 2015-09

**Authors:** Chin Lik Tan, Jessica C.F. Kwok, Janosch P.D. Heller, Rongrong Zhao, Richard Eva, James W. Fawcett

**Affiliations:** John van Geest Centre for Brain Repair, Department of Clinical Neurosciences, University of Cambridge, Cambridge CB2 0PY, UK

**Keywords:** Integrins, Axon growth, Axon regeneration, Talin, Kindlin, Cytoskeleton, Cell adhesion

## Abstract

Integrin function is regulated by activation involving conformational changes that modulate ligand-binding affinity and downstream signaling. Activation is regulated through inside-out signaling which is controlled by many signaling pathways via a final common pathway through kindlin and talin, which bind to the intracellular tail of beta integrins. Previous studies have shown that the axon growth inhibitory molecules NogoA and chondroitin sulfate proteoglycans (CSPGs) inactivate integrins. Overexpressing kindlin-1 in dorsal root ganglion (DRG) neurons activates integrins, enabling their axons to overcome inhibitory molecules in the environment, and promoting regeneration in vivo following dorsal root crush. Other studies have indicated that expression of the talin head alone or with kindlin can enhance integrin activation. Here, using adult rat DRG neurons, we investigate the effects of overexpressing various forms of talin on axon growth and integrin signaling. We found that overexpression of the talin head activated axonal integrins but inhibited downstream signaling via FAK, and did not promote axon growth. Similarly, co-expression of the talin head and kindlin-1 prevented the growth-promoting effect of kindlin-1, suggesting that the talin head acts as a form of dominant negative for integrin function. Using full-length talin constructs in PC12 cells we observed that neurite growth was enhanced by the expression of wild-type talin and more so by two ‘activated’ forms of talin produced by point mutation (on laminin and aggrecan–laminin substrates). Nevertheless, co-expression of full-length talin with kindlin did not promote neurite growth more than either molecule alone. In vivo, we find that talin is present in PNS axons (sciatic nerve), and also in CNS axons of the corticospinal tract.

## Highlights

• Full length talin can activate integrins and stimulate axon regeneration on inhibitory CSPGs.
• The talin head domain can activate integrins, but acts as a dominant negative for talin function.
• Talin and kindlins do not have additive effects on axon growth.
• Talin is transported into the axons of the sciatic nerve and the corticospinal tract.

## 1 Introduction

Integrins are αβ heterodimeric transmembrane molecules found on the surface of many different cell types that interact with extracellular matrix glycoproteins. In the nervous system, they are involved in cell migration, axon growth, synaptogenesis and axon regeneration (Eva et al., 2012a; Lemons and Condic, 2008; Winograd-Katz et al., 2014). Integrin function is regulated in various ways, including inside-out signaling, in which binding of molecules to the intracellular domain can switch the molecules from a low ligand-binding affinity state to a high affinity one (Hynes, 2002). Integrin–ligand binding depends on the affinity state, and subsequently allows the activation and propagation of intracellular outside-in signaling. Enhancing integrin activation promotes axon growth from cultured neurons (Ivins et al., 2000; Lein et al., 2000; Lemons and Condic, 2008; Tan et al., 2011), even in the presence of growth-inhibitory substrates such as chondroitin sulfate proteoglycans (CSPGs) and amino-Nogo (Hu and Strittmatter, 2008; Tan et al., 2011).

Integrin activation is affected by many signaling pathways, whose actions converge onto two families of proteins, talin and kindlins, which interact with the β-integrin cytoplasmic tail at two distinct sites. Talin is a large protein comprising a long C-terminal flexible rod domain (~ 220 kDa) that interacts with F-actin and vinculin while the N-terminal head (~ 50 kDa), contains an atypical four point one protein, ezrin, radixin and moesin (FERM) domain that binds to integrin cytoplasmic tails (Kim et al., 2011; Critchley, 2009; Ye et al., 2014; Calderwood et al., 2013). Binding of the talin head to integrin was identified as a final common step required for integrin activation (Goult et al., 2013; Tadokoro et al., 2003), and overexpression of the head domain is sufficient to induce integrin activation (Calderwood et al., 1999; Kim et al., 2003). Kindlins also associate with the cytoplasmic tail of beta integrins, promoting activation and clustering (Ye et al., 2013, 2014; Calderwood et al., 2013) and recent data suggest that kindlins promote integrin clustering thereby increasing the avidity of integrins for ligands (Ye et al., 2014). Our previous work has shown that expression of kindlin-1, which is not normally expressed in neurons, promotes integrin activation and axon regeneration in the spinal cord (Tan et al., 2012). Kindlin-1 influences Wnt and TGFbeta signaling in addition to its direct effects on integrins (Rognoni et al., 2014). Furthermore, co-expression of the talin head with kindlin-2 results in a synergistic enhancement of integrin activation, as observed in αIIbβ3-expressing CHO cells (Ma et al., 2008; Montanez et al., 2008). Coupled with our previous observation that overexpression of kindlin-1 promotes axon regeneration over inhibitory substrates in vitro and in vivo (Tan et al., 2012), these findings make talin an attractive candidate for promoting axon regeneration.

Here we have investigated the effects of talin and the talin head domain on axon growth and the integrin signaling pathway, either singly or in combination with kindlin-1 or kindlin-2. In addition, we compared the in vivo distribution of talin molecules within the central and peripheral nervous systems.

## 2 Materials and methods

### 2.1 Dorsal root ganglion (DRG) neuron culture

DRGs were dissected from Sprague–Dawley rats (~ 3 months). The neurons were collected, dissociated with collagenase and trypsin, rinsed in calcium- and magnesium-free phosphate-buffered solution (PBS), transfected with expression constructs encoding GFP or talin head–GFP, and plated onto laminin (1 μg/ml) or aggrecan–laminin (25 μg/ml:1 μg/ml) in DMEM supplemented with insulin–transferrin–selenium (1 ×), penicillin–streptomycin–fungizone (1 ×) and 10 ng/ml nerve growth factor.

### 2.2 PC12 cell culture

PC12 cells were plated on collagen IV-coated T75 tissue culture flasks in Roswell Park Memorial Institute (RPMI)-1640 medium supplemented with 10% fetal calf serum, l-glutamine (25 mM) and penicillin–streptomycin–fungizone (1 ×). Neuronally-differentiated PC12 cells were prepared by adding NGF (100 μg/ml) to the culture medium, and incubated for another 10–14 days. Differentiated neurons were transfected with GFP, talin head, talin-1 (full-length), talin-1 (with T1767E mutation) and talin-1 (with E1770A mutation) DNA constructs using the Neon Transfection System (Invitrogen), and plated onto coverslips pre-coated with laminin (1 μg/ml) or aggrecan–laminin (25 μg/ml:1 μg/ml) in the aforementioned culture medium (with NGF).

### 2.3 Transfection with DNA constructs

Transfection was performed using a Neon Transfection Kit (Invitrogen), following the manufacturer's instructions. The transfection parameters used were: DRG neurons, 1200 V, 2 pulses and 20 ms pulse duration and PC12 cells, 1410 V, 1 pulse, and 30 ms pulse duration. The constructs used were kindlin-1 and kindlin-2 linked to cherry donated by Reinhard Fassler (Ussar et al., 2008; Montanez et al., 2008), full length and constitutively active full length talin from David Critchley (Goult et al., 2009) and talin head from Mark Ginsberg (Calderwood et al., 1999). Co-transfection rates using these constructs and methods are around 80% in our laboratory.

### 2.4 Post-fixation immunocytochemistry

Cell cultures on coverslips were fixed with 4% paraformaldehyde (PFA), permeabilized with 0.1% Triton X-100, blocked with goat or donkey serum and incubated with primary antibodies at 4 °C overnight. They were then washed and incubated with secondary antibodies for 1 h at room temperature before being mounted on slides. Primary antibodies were used against beta III tubulin (Sigma, 1:400), pY397 FAK (BD Biosource, 1:100), talin (clone 8D4, Abcam, 1:20), GFP (Abcam, 1:500), and mCherry (Clontech, 1:200).

### 2.5 Live immunocytochemistry

The 9EG7 antibody was added to the cultures for 15 min at 37 °C. After washing with culture medium, the cultures were fixed with 4% PFA. They were then incubated in the FITC-conjugated goat anti-rat antibody for 1 h, before being mounted on slides.

### 2.6 Post-fixation immunohistochemistry of cryostat sections

Animals were first sacrificed with an overdose of sodium pentobarbital, and perfused transcardially with PBS followed by 4% PFA, before dissecting out the spinal cord and sciatic nerve. The tissue was cryoprotected in 30% sucrose solution at 4 °C for 3 days, embedded in OCT (RALamb UK) and cut longitudinally using a cryostat into 14 μm sections. Sections were washed in PBS (15 min, room temperature), permeabilized with 0.1% Triton X-100 (15 min, room temperature), blocked with 10% goat serum (1 h, room temperature) and incubated in primary antibodies (diluted in blocking solution) at 4 °C overnight. The following day, sections were washed with PBS, incubated with secondary antibodies (1 h, room temperature) and then mounted in Fluorosave. Primary antibodies were used against: talin (clone 8D4, Abcam, 1:20) and beta III tubulin (Sigma, 1:400).

### 2.7 Axon growth assay

For DRG neurons, two parameters were quantified as a measure of axon growth, i.e. (a) percentage of neurons with axons longer than the cell body diameter and (b) average of the longest axons extended by each neuron. For PC12 cells, only the axon length was quantified.

### 2.8 Quantitative immunofluorescence (QIF)

At least 20 axons per coverslip were first selected at random and imaged. An area of axons (> 30 μm long) was then traced, and the fluorescence intensity of immunostaining analyzed using the Leica Application Suite (Leica Microsystems).

## 3 Results

### 3.1 Talin head overexpression does not affect axon growth

Talin and the kindlins are the main mediators of inside-out integrin signaling (Karakose et al., 2010; Goult et al., 2009; Kim et al., 2011; Calderwood et al., 2013; Ye et al., 2014). Overexpression of the head domain alone is sufficient to induce integrin activation (Calderwood et al., 1999; Kim et al., 2003). Activation of integrins by manganese, antibodies and kindlin-1 promotes axon growth (Hu and Strittmatter, 2008; Ivins et al., 2000; Lein et al., 2000; Tan et al., 2011). We therefore tested the effect of talin head domain overexpression on axon growth from cultured neurons growing on permissive and inhibitory substrates.

**Fig. 1:**
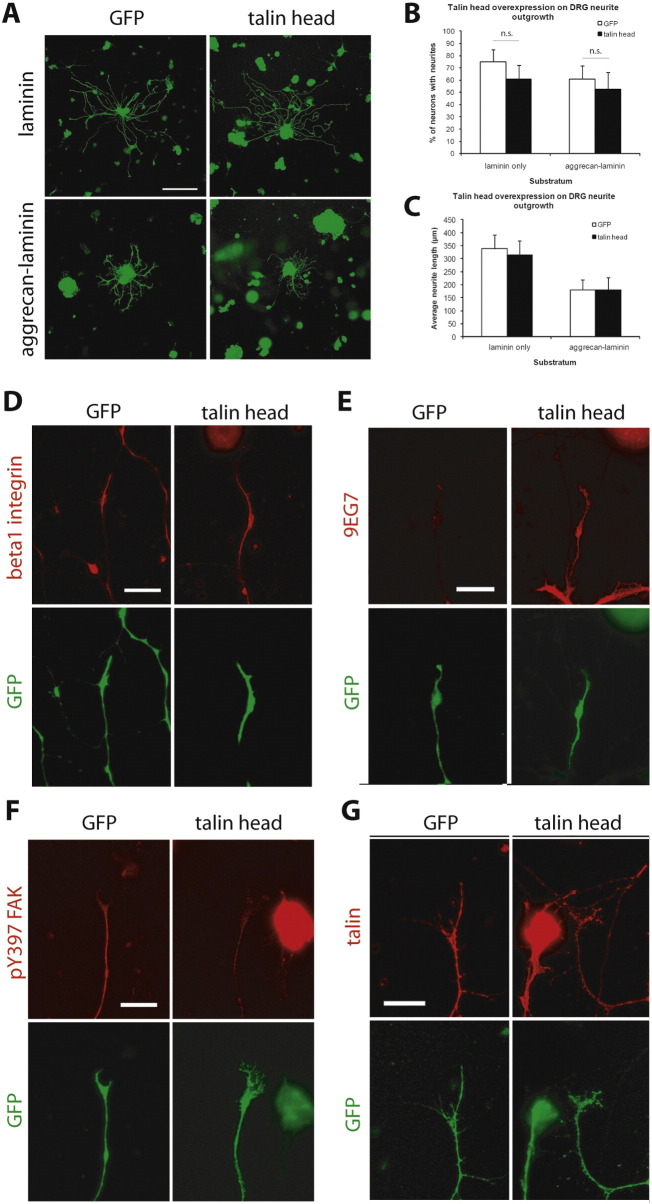
Effects of talin head overexpression on axon growth and integrin function. A–C, Talin head overexpression has no effect on axon growth from cultured DRG neurons. Scale bar, 200 μm. D–G, Talin head overexpression does not affect total quantity of surface β1 integrin (D), enhances ‘inside-out’ integrin signaling as seen through staining with the integrin activation-specific antibody 9EG7 (E), but inhibits ‘outside-in’ signaling via activation of FAK (F), without altering the total expression of full-length talin (G). Data are mean ± s.e.m., and analyzed with Student's t-test. n.s., not significant.

Dissociated adult DRG neurons were transfected with DNA constructs encoding the talin head or control GFP and cultured on growth-promoting (laminin) or growth-inhibitory (aggrecan–laminin) substrates for two days. Surprisingly, overexpression of the talin head did not affect axon growth on either substrate (Fig. 1A–C). We therefore asked if it had had any effect on the activation, expression level or signaling from integrins in the axons. While the total amount of surface β1 integrin was unchanged (p = 0.42, t-test) (Fig. 1D), the level of activated β1 integrin was significantly increased, as measured by 9EG7 immunostaining (+ 30.0%, p < 0.05, t-test) (Fig. 1E). However, this was not accompanied by an increase in the level of ‘outside-in’ integrin signaling, as measured by pY397 FAK intensity. In fact, the pY397 FAK level decreased following talin head overexpression in neurons cultured on laminin (− 48.8%, p < 0.01, t-test) (Fig. 1F). We further determined that overexpression of the head domain of talin did not interfere with the production of endogenous full-length talin by the neurons, as determined using 8D4, an antibody that specifically recognizes the tail domain of talin (Praekelt et al., 2012) (p = 0.81, t-test) (Fig. 1G).

### 3.2 Talin head overexpression reduces the growth-promoting effect of kindlin-1

Previous results suggest that co-expression of the talin head with kindlin produces a synergistic enhancement of integrin activation in αIIbβ3-expressing CHO cells (Ma et al., 2008; Montanez et al., 2008). We therefore asked if this synergistic effect on integrin activation also leads to increased axon growth. DRG neurons were co-transfected with talin head plus either kindlin-1 or kindlin-2 constructs, and axon growth was analyzed after two days.

**Fig. 2:**
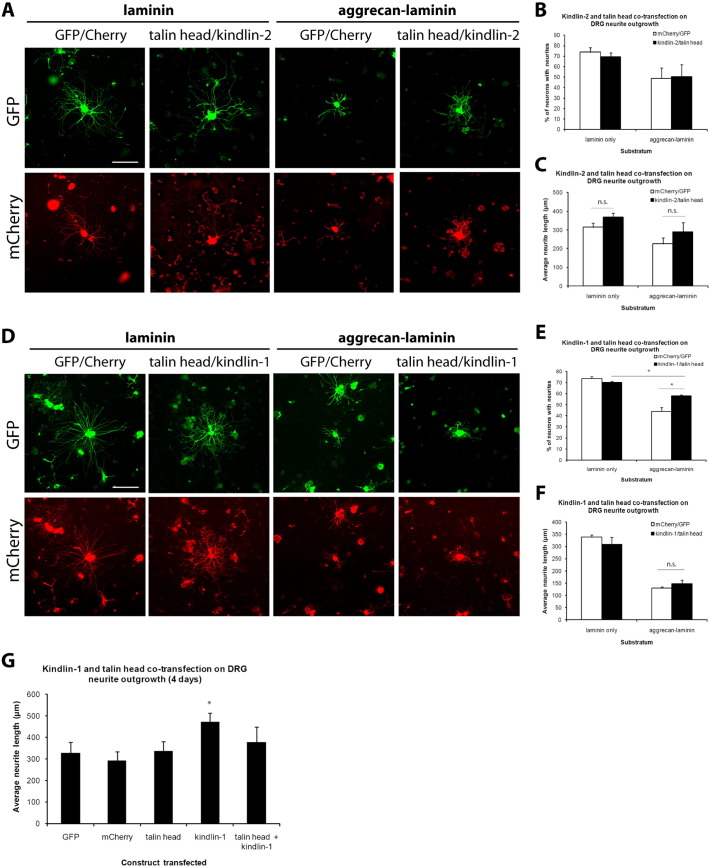
Effects of co-expression of talin head with kindlin-1 or kindlin-2 on axon growth. A–C, Co-expression of talin head with kindlin-2 has no effect on axon growth from cultured DRG neurons, on both growth-promoting (laminin) and growth-inhibitory (aggrecan–laminin) substrates. D–F, Co-expression of talin head with kindlin-1 has a moderate growth-promoting effect on axon growth from DRG neurons cultured on growth-inhibitory (aggrecan–laminin) substratum, when quantified by % of neurons with axons (E). It does not, however, have an effect on neurons cultured on growth-promoting (laminin) substrate. G, Neurons transfected with only kindlin-1 extend longer axons compared to those co-transfected with talin head and kindlin-1 constructs. Scale bar, 200 μm. Data are mean ± s.e.m., and analyzed with Student's t-test or ANOVA for G. *p < 0.05; n.s., not significant.

Co-expression of the talin head and kindlin-2 did not affect axon growth from neurons, whether cultured on laminin or aggrecan–laminin substrates (Fig. 2A–C). Similarly, co-transfection of the talin head and kindlin-1 did not affect axon growth in neurons grown on a growth-promoting substrate (laminin) (Fig. 2D–F). Furthermore, although co-transfection of the talin head and kindlin-1 modestly reversed the inhibition of axon growth by aggrecan (increased percentage of axon-bearing neurons on the aggrecan–laminin substrate, but no effect on axon length), this effect was considerably smaller than what we saw with transfection of kindlin-1 alone, in our previous study (Tan et al., 2012), where there was an almost-complete reversal of inhibition by CSPGs in kindlin-1-transfected neurons. To provide a direct comparison of the effects of kindlin-1 alone with kindlin-1 and talin head in combination, we analyzed growth on aggrecan–laminin substrates after four days (Fig. 2G). Adult DRG neurons were transfected with GFP, mCherry, talin head or kindlin-1–mCherry alone, and co-transfected with talin head and kindlin-1. As in our previous study expression of kindlin-1 alone promoted axon growth, but we now show that co-expression of the talin head with kindlin-1 abolished this effect (Fig. 2G). Expression of the talin head therefore blocks the growth-promoting effect of kindlin-1.

### 3.3 Expression of full-length talin

Many of the effects of talin are mediated by the rod domain. In view of the observation that the talin head was acting as a dominant negative for axon growth, we asked if expressing full-length talin, with the rod domain still intact, has any effect on axon growth. We tried several transfection methods to try to obtain the expression of the talin construct in primary DRG neurons, but were unsuccessful, presumably due to the large size of the construct (12,776 bps). However, the construct could be transfected into PC12 cells, and we therefore performed assays using these cells. We have previously performed various integrin-related neurite outgrowth experiments with the same interventions in DRG neurons and PC12 cells, and have obtained very comparable results between the two types of cells (Eva et al., 2010, 2012b). In addition to wild-type talin, we also tested the effect of two constitutively active forms of talin, talin-1 (T1767E) and talin-1 (E1770A), in which point mutations T1767E and E1770A have been introduced to abolish the self-inhibitory folding between the head and rod domains as seen in wild-type talin (Critchley, 2009; Goult et al., 2009). As with almost all cells, PC12 cells express talin, both talin-1 and talin-2 (Impey et al., 2004).

**Fig. 3:**
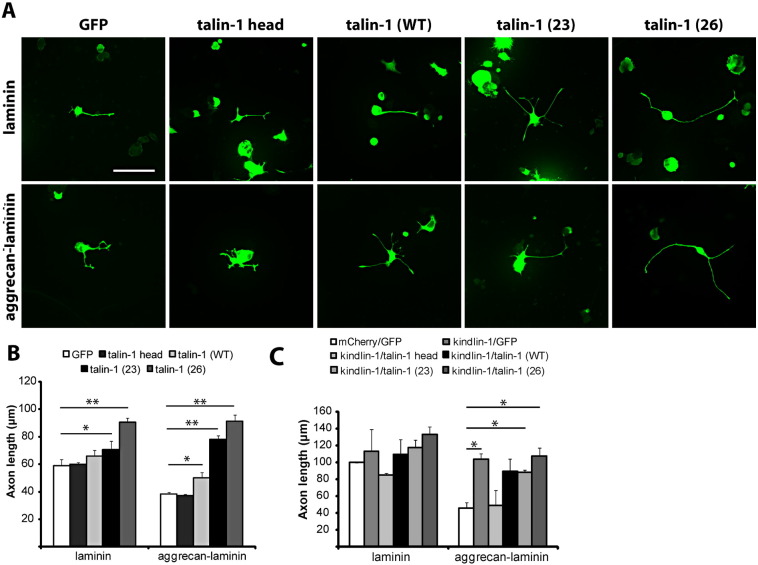
Effects of expression of variants of talin constructs on neurite growth from PC12 cells. A and B, On a growth-promoting (laminin) substrate, expression of talin-1 (T1767E) and talin-1 (E1770A) enhances neurite growth from cultured PC12 cells. On a growth-inhibitory substrate, expression of wild type talin-1, and to a greater extent talin-1 (T1767E) and talin-1 (E1770A) increases neurite growth. Scale bar, 75 μm. C, Co-expression of kindlin-1 with the various talin constructs does not affect axon growth from PC12 cells cultured on laminin-only substrate. On aggrecan–laminin substrate, transfection with kindlin-1 alone, as well as co-transfection of kindlin-1 with talin-1 (T1767E) and talin-1 (E1770A) significantly enhance axon growth, but the effect is not additive. The experiments in B and C were performed with different batches of PC12 cells, hence the difference in control axon lengths. Data are mean ± s.e.m., and analyzed with Student's t-test. **p < 0.01; *p < 0.05; n.s., not significant.

On a laminin substrate on which integrins are strongly activated, overexpression of wild-type talin did not alter axon length (p = 0.52, t-test) (Fig. 3A, B), but expression of activated talin constructs significantly increased axon growth (talin-1 (T1767E), + 19.3%, p < 0.05; talin-1 (E1770A), + 53.4%, p < 0.01, t-test) (Fig. 3A, B). On an inhibitory aggrecan–laminin substrate on which the degree of integrin activation is reduced, overexpression of wild-type talin modestly increased axon growth, while the constitutively active talin-1 (T1767E) and talin-1 (E1770A) had a large effect on axon growth (wild-type, + 31.1%, p < 0.05; talin-1 (T1767E), + 103.6%, p < 0.01; talin-1 (E1770A), + 138.2%, p < 0.01, t-test) (Fig. 3A, B).

We next asked whether expression of talin together with kindlin-1 or kindlin-2 would have a synergistic effect on neurite growth from PC12 cells. When grown on a laminin substrate, none of the full-length talin constructs exhibited a growth-promoting effect when co-expressed with kindlin-1 over and above the effects of the individual molecules alone (Fig. 3C). On inhibitory aggrecan–laminin, we saw, as with DRG neurons, that kindlin-1 expression alone successfully reversed the inhibition of aggrecan (+ 49.1%, p < 0.05, t-test) (Fig. 3C) (Tan et al., 2012). As in the DRG experiments above, expression of the talin head did not increase neurite length. The co-expression of kindlin-1 and the three different types of full length talin had no more effect that the expression of kindlin-1 alone, with no evidence for a synergistic effect (Fig. 3C).

### 3.4 In vivo and in vitro expression of talin in PNS and CNS

**Fig. 4:**
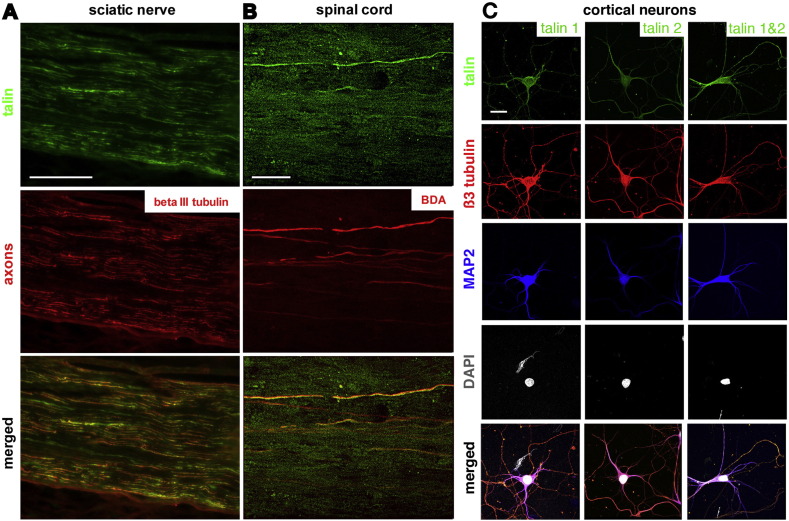
Expression of talin in PNS and CNS. A, Talin is present in beta3-labeled axons and in glia in the rat sciatic nerve (PNS). B, In the dorsal rat spinal cord talin is found in corticospinal tract axons labeled by a cortical injection of BDA. Scale bar, 200 μm.

To further understand the potential role of talin in vivo, we looked at the distribution of endogenous talin in the nervous system, using an antibody (8D4) used in the previous experiments that recognizes both talin-1 and talin-2 (Praekelt et al., 2012). We compared staining in the adult rat sciatic nerve and rat spinal cord. In the sciatic nerve (PNS), the pattern of talin staining was similar to that of beta-3 tubulin-positive axons, although the talin staining was less constant along the axons than tubulin. Also the staining often extended somewhat beyond that of the axons, suggesting the presence of talin in myelin. Occasional glial cells not associated with beta-3 tubulin axons were also stained (Fig. 4A).

In the spinal cord we were particularly interested in the corticospinal tract (CST) because of its relevance to spinal cord repair, and because integrins are excluded from these axons. We therefore stained sections in which CST axons were labeled with BDA following an injection into the sensorimotor cortex two weeks prior to the rats being killed. Most of the corticospinal axons labeled with BDA also showed convincing evidence of talin, although the staining was not bright (Fig. 4B). We also saw talin staining in glia within white matter. In order to verify that cortical neurons express talin, we stained cultured E18 cortical neurons that had been maintained in culture for 7 days with monoclonal antibodies that recognize talin-1 and talin-2 specifically (Praekelt et al., 2012). We were unable to obtain satisfactory staining of tissue sections, but these antibodies revealed that both talin-1 and talin-2 are present in cortical neurons and in all of their processes including axons (Fig. 4C). Integrins are differentially distributed in CNS neurons, being excluded from axons in mature neurons (Franssen et al., unpublished results). However, these results show that if integrin transport into axons is achieved it will not be necessary to also arrange for transport of talin.

## 4 Discussion

### 4.1 Full length talin is necessary for axon growth and integrin signaling

An unexpected finding from these experiments was that talin head overexpression activated integrins but did not enhance axon growth from cultured DRG neurons and PC12 cells, and reversed the growth-promoting effect of kindlin-1; the talin head appeared to be acting as a dominant negative. We confirmed previous observations that talin head overexpression activates integrin (Calderwood et al., 1999; Kim et al., 2003; Tadokoro et al., 2003). Our further analyses of integrin function in transfected neurons suggest an explanation. Talin head overexpression increased the level of activated integrin, assayed through staining with the activation-specific antibody 9EG7, without a change in the total β1 integrin level. This is consistent with previous studies, and with the fact that the talin head contains an integrin-binding PTB site (Calderwood et al., 1999; Kim et al., 2003). Nevertheless, the level of ‘outside-in’ integrin signaling, as assessed by pY397 FAK immunostaining, actually decreased after talin head overexpression, indicating that the head domain alone is not sufficient to substitute for full-length talin in propagating ‘outside-in’ signaling. This corroborates earlier reports that the rod domain of talin is required for proper integrin function. Calpain-II-mediated talin cleavage—which separates the head and rod domains—has been shown to cause FA disassembly (Franco et al., 2004), and expression of the talin head in talin-deficient fibroblasts failed to restore the normal adhesion and migration phenotypes, despite managing to induce integrin activation (Zhang et al., 2008). Conceptually, this explanation is reasonable, since the rod domain contains multiple binding sites for many intracellular proteins, many of which are involved in integrin signaling (Critchley, 2009). These include a binding site for the β integrin cytoplasmic domain, two for actin and 11 for vinculin (Critchley and Gingras, 2008; Calderwood et al., 2013). In addition, the rod domain is necessary for talin dimerization, which may be necessary for downstream signaling (Goult et al., 2013). In the absence of the rod domain, therefore, integrins activated by the talin head are unable to interact with many other adaptor proteins or signaling molecules, hence precluding the transmission of downstream signaling cascades. Moreover, the inability to form connections with intracellular actin may also impede the process of cytoskeletal reorganization necessary for growth cone motility and protrusion during axon extension. Talin links via the FERM domain of its head to the proximal NPXY motif of beta1 integrin, enabling integrin to link to actin via talin. The proximal NPXY motif has many other binding partners, so the talin head might interfere with actin binding and also integrin trafficking through this interaction (Margadant et al., 2012). There are therefore several reasons why an overexpressed talin head might act as a dominant negative for the function of endogenous full-length talin, explaining our result. The likelihood of the talin head having a dominant negative effect is enhanced by the fact that the talin head has a six-fold higher affinity for integrin binding compared to intact talin (Yan et al., 2001).

Viewed in this light, our co-transfection results can be explained. The abolition by the talin head of the growth-promoting effect of kindlin-1 suggests that kindlin-1 is unable to substitute for the functions of the rod domain of talin, and that the dominant negative effect of the talin head can block integrin effects even in the presence of kindlin-1. Co-expression of the talin head and kindlin-2 did not induce increased axon growth because neither would produce such an effect when transfected individually (Tan et al., 2012). Although previous studies reported that co-expression of these two molecules triggered a synergistic enhancement of αIIbβ3 integrin activation in CHO cells (as assayed by the antibody PAC-1), no functional assays such as cell adhesion or migration were performed (Ma et al., 2008; Montanez et al., 2008). Based on our findings, it would be expected that these cells would not exhibit any change in integrin-dependent functions.

### 4.2 Activated talin markedly enhances axon growth

Overexpression of wild type talin in PC12 cells moderately increased axon growth in the presence of inhibitory aggrecan. However two mutated forms of talin that remain in the active configuration produced markedly enhanced growth on both laminin and aggrecan–laminin substrates. These activated talin constructs carry the point mutations T1767E and E1770A, which abolish the self-inhibitory binding between the head (F3) and rod domains (Goult et al., 2009). This suggests that by transfecting the cells with activated talin constructs, the dynamic equilibrium between the activated and self-inhibited talin molecules becomes more directed towards the activated form, resulting in enhanced outside-in integrin signaling and interaction with the cytoskeleton. Surprisingly, co-expression of full-length talin (both wild-type and activated) with both kindlin-1 did not produce any synergistic effect on axon growth. It might have been expected that expression of two integrin activators would lead to enhanced integrin effects. It may be that we are seeing a ceiling effect, with any of the treatments being able to optimize integrin function, or there could be a more complex interaction between the two molecules, as suggested by the effect of kindlins on integrin clustering (Ye et al., 2013).

### 4.3 Two isoforms of talin

There are two forms of talin, talin-1 and talin-2, which differ in gene length, but have similar protein product sizes and share a high degree of amino acid sequence homology (74%). Both isoforms are present in the nervous system, although talin-2 has a higher expression level in the brain (Monkley et al., 2001; Senetar et al., 2007; Debrand et al., 2009). In our experiments, the talin head was that of talin-1. The integrin-binding PTB domains of talin-1 and talin-2 have extremely close sequence similarity (Anthis et al., 2009). In fact, all the crucial residues (R358, A360, Y377) that help create the hydrophobic pocket to accommodate the NPxY motif on β integrin are conserved between the two talin isoforms (Anthis et al., 2009; Garcia-Alvarez et al., 2003). Hence, for our purpose (integrin activation), it is reasonable to conclude that the talin-1 head domain is equivalent to a generic talin head. However, there could be some selectivity of function of the full length molecules. Subcellular localization of talin-1 and talin-2 reveals that talin-1 is found in focal adhesion, linking integrins to the actin cytoskeleton, while talin-2 is present in larger adhesion sites (Senetar et al., 2007; Praekelt et al., 2012). In the invertebrate chordate *Ciona intestinalis*, the two splice variants of the lone talin gene, reflecting talin-1 and talin-2, are also targeted to different sites, with the former at focal adhesions and the latter co-localized with F-actin stress fibers (Singiser and McCann, 2006).

### 4.4 Talin in PNS and CNS axons

Our immunohistochemistry results showed that talin (the antibody recognizes both talin-1 and talin-2) is present in axons and some glia in the sciatic nerve, and could therefore participate in integrin-mediated axon regeneration (Vogelezang et al., 2001; Werner et al., 2000). In the spinal cord we detected talin in corticospinal axons labeled by cortical tracer injections. Integrins are differentially distributed in CNS neurons, being excluded from axons in mature cortical neurons (Franssen et al., unpublished results). However, these results show that if integrin transport into axons is achieved it will not be necessary to also arrange for transport of talin.

## Conflict of interest

James Fawcett is a paid consultant for Acorda Therapeutics and Novartis.
